# Health-Related Quality of Life Is Low in Secondary School Children in Fiji

**DOI:** 10.1155/2012/294530

**Published:** 2012-12-04

**Authors:** Solveig Petersen, Helen Mavoa, Boyd Swinburn, Gade Waqa, Ramneek Goundar, Marjory Moodie

**Affiliations:** ^1^Deakin Health Economics, Faculty of Health, Deakin University, 221 Burwood Highway, Melbourne, VIC 3125, Australia; ^2^Clinical Sciences, Umeå University, 90187 Umeå, Sweden; ^3^WHO Collaborating Centre for Obesity Prevention, Faculty of Health, Deakin University, 221 Burwood Highway, Melbourne VIC 3125, Australia; ^4^Pacific Research Centre for the Prevention of Obesity and Non-Communicable Diseases (C-POND), Fiji School of Medicine, College of Medicine, Nursing and Health Sciences, Fiji National University, Suva, Fiji

## Abstract

The health and wellbeing of children in lower-income countries is the focus of much international effort, yet there has been very little direct measurement of this. *Objective.* The current objective was to study the health-related quality of life (HRQoL) in a general population of secondary school children in Fiji, a low middle-income country in the Pacific. *Methods*. Self-reported HRQoL was measured by the Pediatric Quality of Life Inventory 4.0 in 8947 school children (aged 12–18 years) from 18 secondary schools on Viti Levu, the main island of Fiji. HRQoL in Fiji was compared to that of school-aged children in 13 high- and upper middle-income countries. *Results*. The school children in Fiji had lower HRQoL than the children in the 13 comparison countries, with consistently lower physical, emotional, social, and school functioning and wellbeing. HRQoL was particularly low amongst girls and Indigenous Fijians. *Conclusions*. These findings raise concerns about the general functioning and wellbeing of school children in Fiji. The consistently low HRQoL across all core domains suggests pervasive underlying determinants. Investigation of the potential determinants in Fiji and validation of the current results in Fiji and other lower-income countries are important avenues for future research.

## 1. Introduction

Health-related quality of life (HRQoL) assessment has increasingly been acknowledged as valuable for decision making in clinical and community settings. It provides information about the functioning and wellbeing of a population, identifies population groups with special needs, and can be used to measure intervention impact at a general population level as well as in health care settings [1].

Health-related quality of life is a subjective, multidimensional construct, referring to that part of quality of life which is associated with health, and as such addresses personal perception of physical, emotional, and social functioning and wellbeing [1]. A widely used instrument for HRQoL assessment in children is the Pediatric Quality of Life Inventory, version 4 (PedsQL), which was developed around the year of 2000 by Dr. Varni and colleagues in the USA [2]. The PedsQL is a generic instrument measuring all core aspects of HRQoL, and it has demonstrated acceptable psychometric properties for assessing self-reported HRQoL in general as well as clinical child populations [2–5]. 

In the past, there has been a lack of HRQoL data from child populations, partly due to an absence of standardized paediatric HRQoL instruments, but now some paediatric population level data are available, primarily from high-income countries [5–17]. Despite the international focus on improving the health and wellbeing of children living in lower-income countries, there are no population-based studies of HRQoL in these countries. 

This study measures HRQoL in the Pacific Republic of Fiji, a low middle-income country with a gross national income per capita of $4100 in 2005 US dollars [18]. Its population totalled 837,000 in 2007, with 79% living on Viti Levu, the largest, most populous island, and the site of the capital Suva [19]. Thirty-nine percent of the population were below the age of 19 years and 80% of the 14–18 year olds were enrolled in secondary education [19, 20]. Indigenous Fijians comprised over half of the population (57%), 37% were Indo-Fijians and the rest (6%) belonged to numerous smaller ethnic groups. 

The current paper draws on data from the Pacific Obesity Prevention in Communities (OPIC) study [21]. It aims to investigate the HRQoL in a general population sample of secondary school children in Fiji in terms of age, gender, and ethnicity and to compare the overall results with the HRQoL of school-aged children from other parts of the world. The study will test the null hypothesis that there are no differences in HRQoL between groups.

## 2. Material and Methods 

### 2.1. Study Design and Procedure

The OPIC study, an intervention study focusing on population level prevention of obesity, surveyed 18 secondary schools on Viti Levu: seven of 10 schools in the Nasinu-Nausori corridor, a periurban area of Suva, and 11 of 35 schools in the regional centres of Lautoka, Nadi, and Sigatoka. The latter schools were selected to match the ethnic distribution in the Nasinu-Nausori corridor sample. All students enrolled in these schools, who provided self- and parental-written consent, were recruited. Data were collected by the research team from Fiji National University (in collaboration with Deakin University, Australia) in three waves between August 2005 and July 2008. Students were provided with standardised instructions before they completed an HRQoL questionnaire in the classroom, using personal digital assistant devices. Sociodemographic data were collected at the same time on paper forms. The current study included baseline data from when the student entered the project; 8947 participants provided HRQoL data (participation rate 73%). Ethics approval was provided by the Fiji National Health Research Committee, the Fiji National Research Ethics Review Committee, and the Deakin University Human Research Ethics Committee.

### 2.2. Measures

HRQoL was measured by the English version of the generic Pediatric Quality of Life Inventory 4.0 (PedsQL) self-report module for 13–18 year olds. English is the official language in Fiji (including in schools), and prior to the current study, the instrument's semantic equivalence and cultural appropriateness in the Fiji context was confirmed in a pilot test with two classroom groups, one in a predominantly Indigenous Fijian school, the other Indo-Fijian.

The PedsQL has 23 items and covers physical, emotional, social, and school functioning and wellbeing [2]. It utilizes a recall period of one month and gives five response choices on an ordinal scale ranging from “never” (0) to “almost always” (5). The responses are reversed scored, and linearly transformed to a 0–100 scale, with higher scores indicating better HRQoL. Mean HRQoL summary scores (all 23 items) are calculated in addition to mean scores for each of the four subscales, plus a mean psychosocial health summary score (the 15 items from the emotional, social, and school scales). Scoring and the handling of missing data were undertaken in accordance with the instrument guidelines [2], which require that 50% of items in a scale be answered for a score to be calculated. Additionally, to meet the requirement of the multidimensionality in the HRQoL construct, the current study only included an HRQoL score if 50% of items were answered in each of the four subscales, and a psychosocial health summary score was only included if 50% of items were answered in each of the three related subscales (emotional, social, and school scales). 

Socio-demographic data were collected on age, gender, ethnic group most identified with (ethnicity), number of people usually living in the student's home during the school week (household size), and the student's living arrangements during the school week (family structure).

### 2.3. Comparison Studies from the Literature

A comparison sample of community studies, measuring HRQoL by the PedsQL in school-aged children, was searched in the PubMed and PsycInfo databases (December 2011). When data were available from several populations or age groups within a country, the study with the closest comparability to the Fiji sample, and which was the most representative for that country, was selected. Within this sample, studies were identified in which HRQoL was stratified according to reported health. Additionally, two publications were identified which summarised studies of HRQoL in clinical samples of children (HRQoL measured by the PedsQL) [16, 22]. 

### 2.4. Data Analysis

The Predictive Analytics Software (PASW statistics) version 18 was used to perform statistics. Summary statistics were calculated for all PedsQL scales in the Fiji sample and mean scores were descriptively compared to PedsQL scores from community samples in other countries (whole population and subpopulation with chronic illness) and to scores from clinical populations. Internal consistency of the PedsQL scales was estimated by Cronbach's coefficient alpha, with values ≥0.70 considered acceptable for group comparisons and values ≥0.90 acceptable for individual comparison [23]. The Mann-Whitney *U* test was used to detect HRQoL differences between socio-demographic groups, that is, age group: 12–14 versus 15–18 years; gender: girls *versus* boys; ethnic groups: Indigenous Fijian, Indo-Fijian, and other ethnicities (others); household size: 1–5 versus 6–16 persons. HRQoL differences in age and ethnic subgroup were tested in the total group and by gender. Associations between HRQoL and age group, gender, and ethnicity, were also tested in univariate and multivariate logistic regression models, with HRQoL scores in the highest-lowest quartile as the dependent variable and age, gender, ethnicity, household size, and living arrangements as independent variables. Level of significance was set at 0.05, and the size of difference was estimated by Cohen's *d *with values ≥0.2 considered meaningful but small, and values ≥0.5 and ≥0.8 of medium and large sizes, respectively [24]. 

## 3. Results

### 3.1. Participant Characteristics

The majority of the 8947 participants in Fiji were between 14 and 17 years old, with slightly more girls than boys and more Indo-Fijians than Indigenous Fijians (Table 1). Most participants lived with their parents in households which comprised on average five to six members (Indigenous Fijian 6.4 (SD 2.4); Indo-Fijian 5.1 (SD 1.8); others 6.2 (SD 2.8)). The majority of the households with less than six members were Indo-Fijian (28% Indigenous Fijian; 67% Indo-Fijian; 5% others). On the contrary, Indigenous Fijian households were predominant amongst the larger-size households with 6–16 members (56% Indigenous Fijian; 37% Indo-Fijian; 7% others).

### 3.2. Instrument Properties

None of the students “almost always” experienced all of the 23 studied HRQoL related problems, that is, there was no floor effect, and less than 1% reported that they never experienced any HRQoL related problems, that is, ceiling effects were overall negligible (Table 2). However, about one in five reported the highest possible social functioning and wellbeing, indicating some ceiling effect for this domain. All PedsQL scales showed internal consistency reliability that was acceptable for group level comparisons.

### 3.3. Health-Related Quality of Life

Half of the school children frequently (often or almost always) experienced at least one type of HRQoL problem, and one in four reported that they frequently experienced two or more problems, within at least one of the HRQoL domains (Table 3). 

#### 3.3.1. Age

Younger students (12–14 years) reported slightly lower physical and social, but higher emotional and school functioning and wellbeing than older students (15–18 years) (Table 3). School functioning and wellbeing were highest among the younger age group independent of gender, but other than that, HRQoL followed different patterns across age groups in boys and girls. Younger boys reported 4-5% lower physical and social functioning and wellbeing than older boys (mean (SD) physical: 78.9 (18.8) versus 83.4 (15.7); Cohen's *d* 0.3 social: 77.2 (19.2) versus 80.8 (17.3); Cohen's *d* 0.2). In contrast, older girls reported 5-6% lower emotional and school functioning and wellbeing than younger girls (emotional: 58.7 (16.2) versus 61.7 (17.7); Cohen's *d* 0.2 school: 68.3 (16.0) versus 72.4 (17.8); Cohen's *d* 0.2 both).

#### 3.3.2. Gender

Girls experienced a small-to-medium size lower HRQoL than boys (Table 3), and the odds of being among the 25% with lowest HRQoL were 3 times higher in girls than boys, independent of age, ethnicity, household size, and family structure (Table 4). All aspects of HRQoL were lower in girls than boys, and differences approached medium size with regard to physical and emotional aspects (Table 3). These gender differences were generally consistent across age and ethnic subgroups (not shown in table), but accentuated in older school children (HRQoL in boys-girls ≥15 years, mean (SD): 76.5 (12.5) versus 70.5 (12.2); Cohen's *d* 0.5).

#### 3.3.3. Ethnicity

Indigenous Fijian students experienced lower HRQoL than both Indo-Fijians and those of other ethnicities (Table 3). The odds of being among the 25% with lowest HRQoL were twice as high for Indigenous Fijians compared to Indo-Fijians and others, independent of gender, age, household size, and living arrangements (Table 4). All studied aspects of HRQoL, but especially social and school functioning and wellbeing, were lower in Indigenous Fijians than Indo-Fijians.

#### 3.3.4. Household Size

Students who lived in smaller (1–5 person) households during the school week reported higher HRQoL than students who lived in larger households, primarily due to better social and school functioning and wellbeing (Table 3).

#### 3.3.5. International Comparisons

The participants in the community-based-comparison sample comprised school-aged children in 13 countries in North and South America, Europe, the Middle East, Asia, and Australia [5–17], and three studies [7, 13, 17] provided HRQoL data by parent-rated chronic illness (Table 5). The participants in the clinical comparison groups were school-aged children in the USA, UK, the Netherlands, and Australia, who were diagnosed with asthma, cancer, cardiac disease, cerebral palsy, diabetes, end-stage renal disease, gastrointestinal disease, irritable bowel disease, obesity, psychiatric disorders, or rheumatic disease [16, 22].

**Figure 1:**
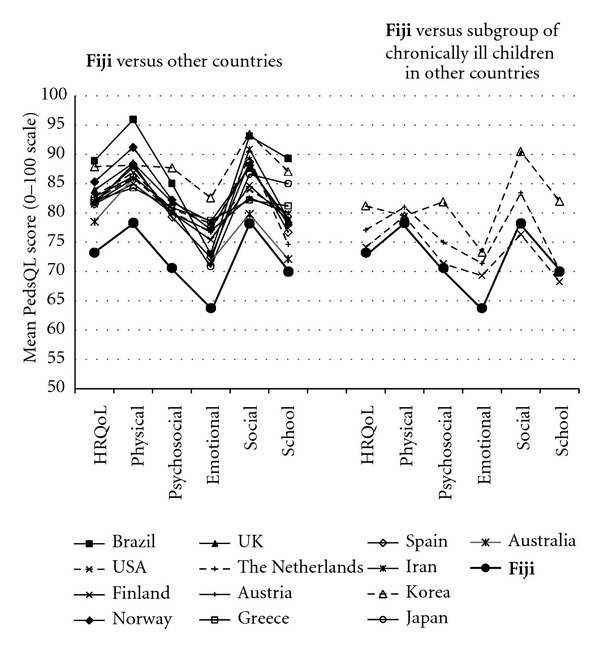
Comparison of self-rated health-related quality of life (HRQoL) and subdomains of HRQoL between community-based samples of school-aged children in Fiji and other countries. Self-rated HRQoL is also compared between the children in Fiji and subgroups of chronically ill children (parent reports) in three countries, namely, USA, Netherlands, and Korea. HRQoL is measured by the PedsQL in all samples. Adapted from [5–17].

**Figure 2:**
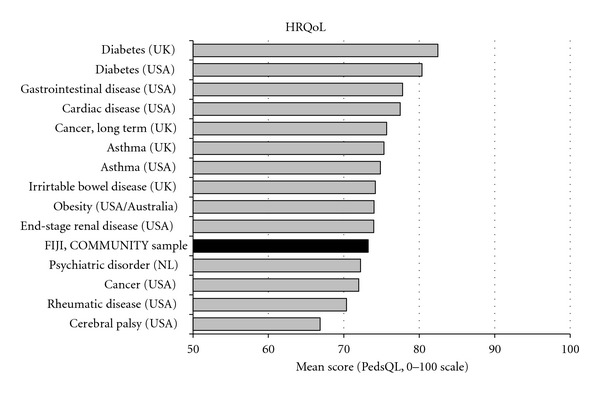
Comparison between self-rated health-related quality of life (HRQoL) in a community sample of school-aged children in Fiji and HRQoL in school-aged patients with specific diagnoses in USA, Australia, UK, and the Netherlands (NL). HRQoL is measured by the PedsQL in all samples. Adapted from [16, 22].

The school children in Fiji had a mean PedsQL score of 73 (SD 13) on the HRQoL scale (Table 2), which was 5 to 15 points lower than in any of the 13 community-based comparison countries (mean (SD) difference: 10 (3)) (Figure 1). The Fiji students' functioning and wellbeing followed the same general pattern as in most of the comparison countries, with emotional and school functioning and wellbeing being lower than physical and social functioning and wellbeing. However, within all studied aspects of HRQoL, the Fiji school children experienced lower functioning and wellbeing than those in the other countries. HRQoL in the Fiji was also lower than, or similar to, that of community-based subgroups who, according to their parents, had chronic illnesses (Figure 1), and lower than the HRQoL in school-aged children with clinical diagnoses of asthma, diabetes, long-term cancer, cardiac disease, end-stage renal disease, gastrointestinal disease, irritable bowel disease, and obesity, but higher than that of children with psychiatric disorders, cerebral palsy, and cancer (not restricted to long term) (Figure 2).

## 4. Discussion 

This study presents data on HRQoL in a large community sample of 12–18-year-old secondary school children in Fiji, who experienced lower HRQoL and consistently lower physical, emotional, social, and school functioning and wellbeing, than children in a range of other countries from around the world. While the school children in Fiji experienced lower HRQoL across age groups, gender, and ethnicity, HRQoL was particularly low in girls (specifically with regard to physical and emotional functioning and wellbeing) and Indigenous Fijians (primarily due to lower social and school functioning and wellbeing). 

The current study and all comparison studies are population based, include school-aged children, and rely on self-reported HRQoL, measured by the PedsQL. However, there are several methodological differences between these studies, for instance, with regard to study design, sample selection, and mode of data collection. HRQoL differences between individual studies may be influenced by such methodological issues, and we therefore do not report on detailed one-by-one comparisons between countries. What is notable, however, is the overall pattern of lower HRQoL in the Fiji sample compared to the HRQoL responses from these other methodologically diverse samples. 

The PedsQL has not yet been comprehensively validated in the Fiji context. However, the consistent evidence of reliability and validity across countries in other parts of the world [2, 4–7, 9, 14–16] indicates that the instrument is stable for use across cultures. This is further supported by tests in the USA showing that the questionnaire is interpreted in similar manners across ethnic subgroups, including Pacific Islanders [25]. In the current study, we confirmed semantic equivalence of the instrument in the two major ethnic groups in Fiji, as well as internal consistency reliability, and we found no severe floor or ceiling effects, which together supports acceptable psychometric properties of the PedsQL in Fiji. 

The age group studied herein includes early-to-late adolescence, an unsettled life stage where some physical, emotional, and social problems may be regarded as part of the natural developmental process [26]. However, a very high proportion of the Fiji students experienced HRQoL problems on a regular basis and HRQoL equal to or below that of clinical populations with a variety of medical conditions. In parallel, the 2010 nationally representative Fiji Global School-based Student Health Survey (GSHS) reported that nearly every fifth (17%) of the 13–15 year olds in Fiji had seriously considered committing suicide during the previous 12 months [27]. Puberty and adolescence is a critical period for brain development, development of a stable self-identity, and for attaining basic skills and knowledge required in adulthood [26]. Low physical, emotional, social and school functioning and wellbeing may inhibit these processes and thereby, not only cause immediate suffering, but may also have negative implications for future life and performance and for the functioning of the society at large. Together, this suggests that the HRQoL of school children in Fiji is of concern.

This study cannot establish causal pathways. However, the consistently low HRQoL across all domains and socio-demographic subgroups suggests that underlying causes are likely to be multifactorial and pervasive. It is beyond the scope of this paper to debate all potential explanations, but some could be related to underlying socioeconomic and/or sociocultural factors.

Fiji is a low middle-income country, with one in four households living in poverty, while the comparison countries are upper middle- or high-income countries [18, 20]. In general, poor health and wellbeing are more prevalent in lower- than higher-income countries, and children living in economic hardship face increased risk of a range of health problems that may lead to, or are directly related to compromised physical, emotional, and social functioning, and wellbeing (e.g., infectious diseases; mood, conduct and hyperkinetic problems) [28, 29]. Also, income inequality is relatively high in Fiji, exceeded only by Brazil among the comparison countries [18, 20], and distribution of wealth may negatively influence different aspects of HRQoL. For instance, at a population level, high income inequality is associated with paediatric overweight, mental health problems including emotional and behavioural problems, impaired peer relationships and bullying, and also lower academic performance [30].

Taken together, a poor economy coupled with wide income inequality may have the potential to impact negatively on all core aspects of HRQoL. This is supported by several studies from Europe and the USA, which have shown a link between low socioeconomic status at either a family or school level, and impaired functioning and wellbeing across all core aspects of HRQoL in school-aged children [8, 31, 32]. Our study found particularly low HRQoL in large households and among Indigenous Fijians. The Fiji household survey reports that both large households and Indigenous Fijians have poorer economic conditions than their counterparts [20], indicating a potential association between lower economic means and low HRQoL, also in Fiji.

The main social groups that influence children's lives are their family, school, and peers. Child rearing in Fiji follows the principles that until recently were customary in most parts of the world, including correcting children by use of corporal punishment, a practice with growing evidence of troublesome side effects [33–37]. An extensive review from 2002 showed that children who received corporal punishment, more often experienced physical ill-health and injuries, short- and long-term externalizing and internalizing mental problems, and impaired relations with parent and peers [35]. Furthermore, corporal punishment may impact negatively on cognitive development and academic performance [36, 37]. While some countries, including six of the current comparison countries, had ratified a total ban on corporal punishment at the time of our study [38], corporal punishment was still common practice in homes and schools in Fiji, and there is evidence that this practice imposes negatively on the functioning and wellbeing of school-aged children in Fiji [34, 39].

Bullying can also impact negatively on HRQoL [40, 41] and this type of interaction seems relatively common among school-aged children in Fiji. A recent nationally representative survey in Fiji (the GSHS) reported that 42% of the 13–15-year-old participants had been bullied in the month prior to the survey [27]. In contrast, the eight current comparison countries in Europe and North America participated in similar surveys year 2009/2010, and all reported lower levels of bullying than reported in Fiji for this age group [42], and so did an analogous but earlier survey in Australia [43].

The lower HRQoL in Indigenous Fijian compared to Indo-Fijian students in our study may potentially be explained by socio-cultural differences between the two ethnic groups. Indigenous Fijians have a collectivist ethos, or world view, characterised by interdependence, group needs superseding those of individuals, and expectations that children undertake chores and support social events in the family [44–46]. In contrast, Indo-Fijians have a more individualistic world view that values independence, pro-activity and autonomy and gives higher priority to study and academic achievement [45, 47]. These different world views have been reflected in a more authoritarian parenting style in Indigenous Fijians, with expectations that children obey adults without question and avoid seeking attention to themselves [48]. Indo-Fijian parents are generally more authoritative or permissive, seeking and responding to their children's preferences [44]. The particularly low school functioning in Indigenous Fijians in our study is paralleled by their lower academic success compared with their Indo-Fijian peers [39, 49]. This may partly be explained by the Fiji school curriculum being based on individual achievement and western models and therefore less congruous with Indigenous Fijian world views than those of Indo-Fijians [50]. Further, in contrast to Indo-Fijians, Indigenous Fijian school children may also have to reconcile the cultural differences between family and education settings [51]. However, at large, there is a lack of strong recent evidence to provide explanations for the ethnic differences in HRQoL in the current study. 

Girls reported lower physical and emotional functioning and wellbeing than boys, in line with the literature [6, 12, 15, 16, 52, 53], but the lower social and school functioning and wellbeing in girls in Fiji is puzzling, generally these aspects of HRQoL are similar in boys and girls [6, 16, 52], or possibly better in girls [12]. Lower functioning and wellbeing in adolescent girls has been explained by biological differences and differences in gender roles and gender socialization [26, 42, 52]. Biological sex differences such as earlier onset and greater intrusiveness of puberty and brain development in girls are universal, while gender roles and socialization are culture dependent. There is some evidence that girls in Fiji do more chores than boys [44, 54], and that in the case of Indo-Fijians this is associated with lower academic performance in girls, but at the same time, Indo-Fijian parents have higher expectations of girls' school performance [54]. Thus, expected gender roles may also contribute to lower functioning and wellbeing in girls in Fiji. Other than that, there is limited solid evidence to underpin explanations of the consistently lower HRQoL in girls in Fiji.

Additionally, Fiji has undergone multiple transitions in the last decades, including a rural-urban shift. In 2008, 49% of Fiji citizens lived in urban areas, a 16% increase from 2002 [20]. Indo-Fijians moved from rural areas when their land leases expired, while Indigenous Fijian families moved from outlying islands to the main urban centres for educational reasons [50, 55]. Studies in other countries suggest that for school-aged children, urbanisation per se, as well as moving to a new environment, is associated with higher levels of physical and emotional problems and also exposure to high risk social environments and development of high risk behaviours [56, 57]. Concurrently, the traditional cultural world views of the ethnic groups in Fiji have been challenged by increased exposure to western values and expectations, especially via the media, including television which was introduced in 1995, commerce, and tourism [58]. Living in this type of environment with exposure to contradictory values is stressful for school-aged children [59] and may have contributed further to the low HRQoL seen in the students in the current study.

The current study was unique, being the first to report on HRQoL in children in the Pacific region. Other strengths of the study were the large population size, including 13% of all students enrolled in secondary schools in Fiji [60], and also the use of a standardized measure of HRQoL which has been widely utilized around the world. With regard to the nonprobabilistic sampling procedure, which is a potential limitation, our sample mirrored secondary school children in Fiji with regard to gender distribution, but had a higher representation of Indo-Fijians (53% versus 40%), and a larger average household size than in the whole of Fiji (5.7 versus 4.8 persons) [20, 60]. Note, in terms of HRQoL on an overall population level, these two potential sample biases would tend to cancel out each other. Also, the overall incidence of poverty in the area of the study was similar to that of the Fiji at large [19]. Thus, there are reasons to believe the current results are likely to be similar to that of secondary school children in Fiji in general, but further testing is needed to validate this assumption. Additional validation of the PedsQL in Fiji is also warranted, specifically with regard to content validity and item functioning across cultures.

In conclusion, This study presents population data indicating markedly low HRQoL in secondary school children in Fiji, with low functioning and wellbeing across all four core aspects of the HRQoL and particularly in the case of girls and Indigenous Fijians. These findings may have important implications at both an individual level and for the Fiji society at large. While potential explanations have been proposed for these findings, the supporting evidence is limited. Because this is the first study of school children's HRQoL in this geographical region and in a lower-income country, substantially more research is needed to verify the findings in Fiji and other lower-income-countries, and also to explore in more depth the potential explanations and solutions.

## Figures and Tables

**Table 1:** Basic characteristics of the study participants.

	Participants	
	*N *	%
Sex		
Girls	4747	53.1
Boys	4200	46.9
Total	8947	
Age (years)		
12	8	0.1
13	789	8.8
14	1949	21.8
15	1626	18.2
16	2314	25.9
17	1508	16.8
18	753	8.4
Mean age 15.95 (SD 1.41)		
Ethnicity		
Indigenous Fijian	3725	41.6
Indo-Fijian	4713	52.7
Others	509	5.7
Number in household		
1–5 persons	4666	52.2
6–10 persons	3609	40.3
11–16 persons	276	3.1
Unknown	396	4.4
Mean persons/household 5.69 (SD 2.24)		
Family situation during school week		
Live with 2 parents	6335	70.8
1 parent	1162	13.0
Other adult relatives	931	10.3
At boarding school	318	3.6
None of the above	121	1.4
Unknown	80	0.9

SD: standard deviation.

**Table 2:** Self-rated health-related quality of life (HRQoL) and subdomains of HRQoL in 12–18-year-old school children in Fiji.

	*N *	Mean (SD)	Percentiles			Min-max	Percent				Cronbach's *α*
			25	50	75		Floor	Ceiling	Often or almost always		
									≥1 problem	≥2 problem	
HRQoL	8786	73.23 (13.16)	65.22	73.91	82.61	3.26–100	0.00	0.65	51.4	25.0^1^	0.87
Physical	8947	78.31 (16.87)	68.75	81.25	90.63	0.00–100	0.03	7.15	24.6	11.8	0.84
Psychosocial	8786	70.59 (13.96)	60.00	70.00	80.00	5.00–100	0.00	1.10	44.8	19.1^1^	0.84
Emotional	8947	63.73 (17.25)	50.00	60.00	75.00	0.00–100	0.07	3.89	31.6	12.1	0.72
Social	8946	78.22 (18.05)	65.00	80.00	95.00	0.00–100	0.04	19.33	17.0	6.0	0.75
School	8787	69.99 (16.74)	60.00	70.00	80.00	0.00–100	0.17	5.82	19.8	7.3	0.68

SD: standard deviation.

^1^ At least 1 subdomain with at least 2 problems occurring often or almost always, base *n* = 8947.

**Table 3:** Self-rated health-related quality of life (HRQoL) by sociodemography in 12–18-year-old school children in Fiji.

		HRQoL	Physical	Psychosocial	Emotional	Social	School
Sex							
Girls	Mean (SD)	70.79 (12.69)	75.00 (16.21)	68.64 (13.61)	59.63 (16.75)	76.93 (18.02)	69.56 (16.69)
Boys	Mean (SD)	75.98 (13.15)	82.05 (16.83)	72.80 (14.02)	68.36 (16.63)	79.68 (17.98)	70.49 (16.79)
Girls versus boys	*P *	0.000	0.000	0.000	0.000	0.000	0.016
	Girls < boys (%)	6.83	8.59	5.71	12.77	3.45	1.32
	Cohen's *d *	0.40	0.43	0.30	0.52	0.15	0.06

Age							
12–14 y	Mean (SD)	72.94 (14.22)	76.37 (18.20)	71.22 (14.96)	64.95 (17.93)	76.59 (19.06)	72.37 (17.95)
15–18 y	Mean (SD)	73.35 (12.67)	79.17 (16.18)	70.32 (13.49)	63.19 (16.92)	78.94 (17.54)	68.94 (16.07)
12–14 y versus 15–18 y	*P *	NS	0.000	0.003	0.000	0.000	0.000
	Young < old (%)	0.56	3.54	−1.28	−2.79	2.98	−4.98
	Cohen's *d *	0.03	0.16	0.06	0.10	0.13	0.20

Ethnicity							
Indigenous Fijian	Mean (SD)	70.52 (13.43)	76.70 (19.00)	67.24 (13.85)	63.16 (17.43)	73.17 (18.65)	65.41 (16.37)
Indo-Fijian	Mean (SD)	75.26 (12.67)	79.15 (15.20)	73.30 (13.53)	64.17 (17.01)	82.17 (16.70)	73.71 (16.23)
Other	Mean (SD)	74.83 (11.96)	82.33 (13.49)	70.83 (13.31)	63.77 (17.99)	78.61 (16.45)	70.13 (15.72)
Indig.F versus Indo-F	*P *	0.000	0.005	0.000	0.001	0.000	0.000
	Indig.F < Indo-F (%)	6.30	3.10	8.27	1.57	10.95	11.26
	Cohen's *d *	0.36	0.14	0.44	0.06	0.51	0.51
Indig.F versus others	*P *	0.000	0.000	0.000	NS	0.000	0.000
	Indig.F < others (%)	5.76	6.84	5.07	0.96	6.92	6.73
	Cohen's *d *	0.34	0.34	0.26	0.03	0.31	0.29

Household size							
1–5 persons	Mean (SD)	74.40 (12.96)	78.89 (16.21)	72.10 (13.88)	64.58 (17.13)	80.15 (17.71)	71.68 (16.61)
6–16 persons	Mean (SD)	71.92 (13.23)	77.67 (17.52)	68.89 (13.84)	62.57 (17.25)	76.12 (18.22)	68.14 (16.64)
1–5 p versus 6–16 p	*P *	0.000	0.024	0.000	0.000	0.000	0.000
	1–5 p < 6–16 p (%)	−3.33	−1.55	−4.43	−3.10	−5.03	−4.95
	Cohen's *d *	−0.19	−0.07	−0.23	−0.12	−0.22	−0.21

SD: standard deviation.

NS: *P* > 0.05.

Cohen's *d* specifies magnitude of difference between groups; values of 0.20 nominated as small, 0.5 as medium, and 0.8 as large.

**Table 4:** Unadjusted and adjusted odds for health-related quality of life (HRQoL) in the lowest quartile, in sociodemographic subgroups of 12–18-year-old school children in Fiji.

	HRQoL ≤ 25 percentile	Crude OR	95% CI	*P *	Adjusted^1^ OR	95% CI	*P *
Sex							
Reference: Boys	18.2%	1	2.78−3.57	0.000	1	2.50–3.24	0.000
Girls	30.7%	3.15			2.85		

Age Group							
Reference: 15–18 y	23.7%	1	0.91–1.17	0.656	1	0.93–1.24	
12–14 y	27.5%	1.03			1.04		0.617

Ethnicity							
Reference: Indo-Fijian	19.8%	1	2.24–2.89	0.000	1	1.72–2.30	0.000
Indigenous Fijian	31.6%	2.54			1.99		
Reference: Other	20.4%	1	1.76–3.03	0.000	1	1.81–3.24	0.000
Indigenous Fijian	31.6%	2.31			2.42		

OR: odds ratio.

CI: confidence interval.

^1^Model includes: gender, age, ethnicity, household size, and family structure.

**Table 5:** Basic characteristics of community-based comparison groups.

	*N *	Sample/setting	Age (years)	Comment
Brazil [12]	180	Public school children in urban areas of the periphery of greater São Paulo.	5–18	Low income population. No chronic or acute illness 30 days prior to interview.

USA [17]	5972	All new enrollees in State Children's Health Insurance Program in California.	5–16	Representative of low-income families. Chronic disease reported by parents in 574 children (asthma, attention deficit hyperactivity disorder, depression, diabetes etc.).

Finland [14]	1033	All Finnish primary school children in a city of 175.000 inhabitants.	9-10	

Norway [15]	425	Children in 5 junior high schools in 3 urban and 2 rural areas.	13–15	

United Kingdom [16]	1034	Children in 23 schools in South Wales	8–18	Mean age 12.6 years. Children with chronic disease excluded.

The Netherlands [7]	148	Children in 4 elementary schools, 4 high schools, and 1 vocational school in Amsterdam and surrounding region.	13–18	Mean age 15.0 years. Sampling, stratified by geographic location and migrant and parental education in school. Chronic disease reported by parents in 25 children (asthma, congenital defect, skin disease, migraine, etc.).

Austria [8]	1412	Children from 22 schools in Vienna.	8–12	

Greece [9]	645	Representative sample of Greek school children.	8–12	

Spain [10]	511	Representative sample of school children in grades 4–11 in Tarragona.	9–17	Mean age 11.7 years.

Iran [6]	848	Children in 4 secondary and high schools in Tehran.	—	Mean age 15.7 (±1.2)

Korea [13]	1425	Children in 5 elementary, 5 middle, and 4 high schools within 2 small, 2 metropolitan and 1 capital city.	8–18	Chronic disease reported by parents in 50 children.

Japan [5]	922	Children from 1 elementary, 1 middle, and 1 high school in Tokyo.	6–18	

Australia [11]	2890	Children from 13 secondary schools in the Barwon South West region of Victoria.	11–18	Mean age 14.6 years.
